# Social Inequalities of Functioning and Perceived Health in Switzerland–A Representative Cross-Sectional Analysis

**DOI:** 10.1371/journal.pone.0038782

**Published:** 2012-06-12

**Authors:** Jan D. Reinhardt, Erik von Elm, Christine Fekete, Johannes Siegrist

**Affiliations:** 1 Swiss Paraplegic Research (SPF), Nottwil, Switzerland; 2 Department of Health Sciences and Health Policy, University of Lucerne, Lucerne, Switzerland; 3 Institute for Social and Preventive Medicine, University of Lausanne, Lausanne, Switzerland; 4 Institute for Medical Sociology, Heinrich Heine University of Düsseldorf, Düsseldorf, Germany; University of Utah, United States of America

## Abstract

Many people worldwide live with a disability, i.e. limitations in functioning. The prevalence is expected to increase due to demographic change and the growing importance of non-communicable disease and injury. To date, many epidemiological studies have used simple dichotomous measures of disability, even though the WHO's International Classification of Functioning, Disability, and Health (ICF) provides a multi-dimensional framework of functioning. We aimed to examine associations of socio-economic status (SES) and social integration in 3 core domains of functioning (impairment, pain, limitations in activity and participation) and perceived health. We conducted a secondary analysis of representative cross-sectional data of the Swiss Health Survey 2007 including 10,336 female and 8,424 male Swiss residents aged 15 or more. Guided by a theoretical ICF-based model, 4 mixed effects Poisson regressions were fitted in order to explain functioning and perceived health by indicators of SES and social integration. Analyses were stratified by age groups (15–30, 31–54, ≥55 years). In all age groups, SES and social integration were significantly associated with functional and perceived health. Among the functional domains, impairment and pain were closely related, and both were associated with limitations in activity and participation. SES, social integration and functioning were related to perceived health. We found pronounced social inequalities in functioning and perceived health, supporting our theoretical model. Social factors play a significant role in the experience of health, even in a wealthy country such as Switzerland. These findings await confirmation in other, particularly lower resourced settings.

## Introduction

Around one billion people worldwide are disabled, i.e. experience moderate to severe functional limitations [1]. The prevalence of disability is expected to further increase due to factors including demographic change and growing importance of non-communicable diseases and injuries [1], [2], [3], [4], [5]. Although “for the society as a whole, ‘function[ing]’ must be a major effect" (p. 881), comparative effectiveness research and, surprisingly, even disparities research and social epidemiology have rarely made use of comprehensive measures of functioning [6]. Switzerland, as one of the richest countries of the world [7], provides a good example for studying the role of social inequalities in functioning. If we find social inequalities of functioning in a country with a high expenditure on health care and social security [7], we would expect them to be even more pronounced in less wealthy countries.

Several previous epidemiologic studies have shown social gradients of disability [8], [9], [10], [11], [12], [13], [14], [15], [16] and chronic pain [17]. These were, however, mostly confined to the elderly population [8], [9], [10], [11], [12], [13]. Comparative data on younger and middle-aged populations are still lacking. Predictors have usually been comprised of measures of socio-economic status (SES) [12], [13], with indicators of social integration being used less often [8], [16]. So far, studies have assessed disability mainly by dichotomous outcome variables, e.g. asking respondents whether or not they experienced “a long lasting condition that substantially limits one or more basic physical activities [...]" (p. 698) [12]. Even if more complex scales were used, information was often reduced to a binary outcome [1], [13]. In the case of Switzerland, several reports on social inequalities and health are available. Based on data from the Swiss Health Survey 1997, a social gradient of disability was shown in persons aged 65 and older, but younger respondents were not examined [18]. Information on impairments and activity limitations was combined and the outcome was again dichotomized. A report based on the Swiss Health Survey 2007 showed increasing problems in relation to activities of daily living with older age but did not account for SES [19].

Rather than viewing disability as a static attribute of an individual or population, the WHO's International Classification of Functioning, Disability, and Health (ICF) proposes a dynamic and multi-dimensional approach [20], [21], [22], [23]. Disability can be conceptualized as a continuum of problems in functioning, varying across life situations and environments [22]. This concept of functioning encompasses the actual state of body functions and structures, as well as activity and participation (A&P) in a given environment. In a recent study of British women aged 64–83, Dale and colleagues [24] found, for instance, that only restrictions in participation and limitations in complex activities were associated with increased risk of mortality in their fully adjusted models, while impairment was not.

**Figure 1:**
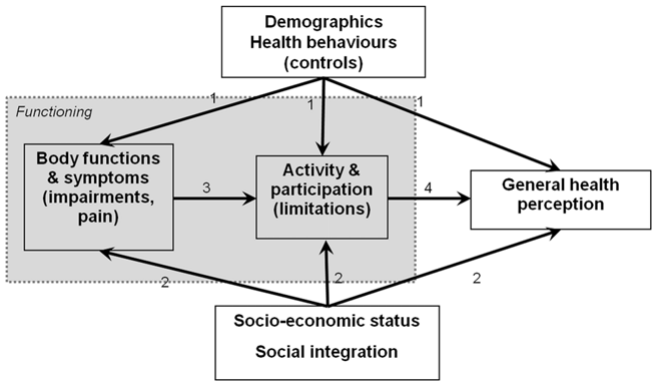
ICF-based theoretical model of social determinants of functioning and perceived health (numbers on edges indicate differential pathways).

Drawing on the ICF framework, we propose a theoretical model (Figure 1) to guide our empirical analysis. While controlling for demographics and health behaviours (pathway 1), we hypothesize (a) that the components of functioning (i.e. impairment, pain, and A&P limitations) are determined by SES and social integration (pathway 2); (b) that A&P limitations are influenced by impairment and pain (pathway 3), and (c) that impairment, A&P limitations, low SES and poor social integration exert a cumulative effect on perceived health [25] (pathway 4).

## Methods

### Design

We analyzed data from the Swiss Health Survey (SHS) 2007 [26] obtained from the Federal Statistics Office of Switzerland (Bundesamt für Statistik: Schweizerische Gesundheitsbefragung 2007). The SHS 2007 adopted the ICF framework by including measures of functioning for population surveys developed by the United Nations' Washington Group on Disability Statistics [27].

### Sample & data collection

The survey was based on a representative stratified random sample of all private Swiss households with fixed line telephones. Household members aged 15 and above were randomly selected. The survey was completed by 18,760 persons, corresponding to a participation rate of 66%. People who did not speak German, French or Italian, were asylum seekers, had an extremely poor health status or lived in nursing homes were excluded [26].

Data were collected with Computer Assisted Telephone Interviews. People aged 75 or above were interviewed face-to-face [26].

### Study variables

#### Functioning

The SHS 2007 included several measures of impairment, pain and A&P limitation with heterogeneous scales of questionnaire items (nominal, ordinal and metric variables). Therefore, we dichotomized all respective information such that a value of 1 indicated the presence of problems and a value of 0 corresponded to none. Following the ICF framework, we then constructed 3 separate sum indices. The *impairment index* comprised problems in the following areas: vision, hearing, speaking, body weight (body mass index <16 kg/m2 or >30 kg/m2), urinary continence, defecation, energy and drive, sleep, and heart rate. The *A&P limitation index* was based on problems in the following activities: walking, eating, getting up from a bed or chair, dressing, toileting, bathing, preparing meals, using a telephone, doing the laundry, finances/accounting, public transport, major household tasks, and shopping. The *pain index* used information on anatomical locations of pain including head, chest, stomach, back, hands and joints. Graphical and statistical tests (using Stata command *nbvargr*) showed that these indices shared a Poisson distribution.

#### Perceived health

Perceived health was measured by asking respondents how they would rate their health in general (ordinal rating scale with the 5 options: very good, good, fair, poor, and very poor).

#### SES

Level of education, income, paid employment, gender and migration background were used as indicators of SES. Education was categorised according to the International Standard Classification of Education as the total number of years of formal education including school and vocational training [28]. Income was measured by net equivalent household income according to OECD criteria [29]. Participants were asked whether or not they were currently in paid employment. Information on migration background was gathered by determining whether at least one parent was of foreign origin.

#### Social integration

We constructed 2 sum indices for social network utilization and perceived social support individually. The social network utilization index encompassed information on whether the following activities were performed at least weekly: visits of family members, phone calls with family members, visits of friends, phone calls with friends, and participation in clubs or associations. The perceived social support index comprised information on four items: having someone to turn to, having at least one supportive family member, feeling lonely (reversely coded), and missing someone to turn to (reversely coded). In addition, we considered whether respondents were married or not.

#### Social class

In order to test for an overall social gradient in disability, we integrated information on income, education and social integration and constructed a social class index with 4 groups. We thus followed Bourdieu's concept of social class representing core dimensions of economic, cultural and social capital [30]. Persons with income, education and a combined social support and network index below average were assigned to the first group (lowest class). People with 2 out of 3 indicators below average were assigned to the second group (low to middle class). Respondents with one indicator below average were assigned to the third class (middle to high class); and those with all indicators above average to the fourth group (highest class). The 2 indices on social integration were combined to avoid over-representation of social capital. The resulting distribution of social classes in the Swiss general population had high face validity with most respondents being assigned to the middle classes and the lowest percentage to the highest class (Table 1).

**Table 1:** Characteristics of the study population.

	Total	15–30 years	31–54 years	≥55 years
*Control Variables*				
Age	49.8 (18.5)	22.9 (4.8)	42.3 (6.5)	68.3 (9.1)
Alcohol in grams per day, mean (SD)	9.3 (15.2)	9.3 (16.7)	8.6 (14.3)	9.9 (15.4)
Current smoker, n (%)	5,091 (27.1)	1,165 (36.7)	2,537 (31.8)	1,389 (18.2)
Low physical activity, n (%)	4,775 (25.5)	548 (17.3)	1,830 (23.0)	2,397 (31.4)
*Functioning*				
Number of pain problems, mean (SD)	1.6 (1.4)	1.5 (1.3)	1.6 (1.4)	1.7 (1.4)
At least one pain problem, n (%)	14,221 (75.8)	2,354 (74.2)	5,988 (75.2)	5,879 (77.1)
Impairment, mean (SD)	1.4 (1.3)	1.1 (1.0)	1.2 (1.2)	1.7 (1.5)
At least one impairment, n (%)	13,232 (70.5)	2,093 (66.0)	5,322 (66.8)	5,817 (76.3)
Number of A&P limitations, mean (SD)	0.4 (1.4)	0.2 (0.8)	0.2 (1.0)	0.8 (1.9)
At least one A&P limitation, n (%)	3,017 (16.1)	278 (8.8)	598 (7.5)	2,141 (28.1)
*Socio-economic status*				
Male, n (%)	8,424 (44.9)	1,535 (48.4)	3716 (46.6)	3,173 (41.6)
Paid employment, n (%)	11,498 (61.3)	2,255 (71.1)	6,852 (86.0)	2,391 (31.4)
Years of formal education, mean (SD)	13.0 (3.5)	12.7 (2.9)	13.8 (3.5)	13.0 (3.5)
Income (CHF), mean (SD)	4,105 (2,979)	3,722 (2,431)	4,140 (3,227)	4,229 (2,901)
Migration background (foreign origin of at least 1 parent), n (%)	5,308 (28.3)	1,005 (31.7)	2,670 (33.5)	1,633 (21.4)
*Social integration*				
Social support (sum index), mean (SD)	3.4 (0.8)	3.3 (0.8)	3.4 (0.8)	3.3 (0.9)
Social network utilization (sum index), mean (SD)	3.2 (1.6)	3.2 (1.1)	3.3 (1.1)	3.0 (1.2)
Married, n (%)	9,550 (50.9)	494 (15.6)	4,889 (61.4)	4,167 (54.7)
*Social class*				
Lowest, n (%)	4,901 (26.1)	974 (30.7)	1,639 (20.6)	2,288 (30.0)
Low to middle, n (%)	7,486 (39.9)	1,320 (41.6)	3,156 (39.6)	3,010 (39.5)
Middle to high, n (%)	4,547 (24.2)	663 (20.9)	2,243 (28.2)	1,641 (21.5)
Highest, n (%)	1,826 (9.7)	215 (6.8)	928 (11.7)	683 (9.0)

Note: A&P  =  Activity and Participation.

### Data analysis

All computations were made with Stata 11 (Stata Corp, College Station, USA) with the exception of the missing value imputation which was performed with R. The indices described above were checked using Stata's confirmatory factor analysis module *confa* [31]. For each index, the index solution was tested against the null hypothesis of a diagonal structure of the covariance matrix (i.e. independence).

To test our hypotheses, we calculated separate mixed effects regressions for the 4 outcomes of impairment, pain, A&P limitation, and perceived health. The 26 Swiss Cantons (territories equivalent to counties) were nested according to language regions (German, French and Italian). Five Cantons are bilingual and in order to obtain a fully nested model were sub-divided into smaller areas according to the dominant language. Random intercepts were introduced for the language region and Canton (or sub-area for the 5 bilingual Cantons) to account for unobserved heterogeneity due to clustering of respondents by language region and Canton. We used Poisson links to model pain, impairment, and activity limitation, and an ordinal probit link to model perceived health, assuming a latent metric variable underlying the ordinal scale distribution [32]. In order to assess the models' fit to the data, we calculated Pseudo R^2^ values by comparing the log likelihood of the full model with that of an empty model.[33] For the Poisson type models, we used the Stata command *xtmepoisson* and for the ordinal probit model, the command *gllamm* [34]. We calculated 4 models for each outcome: an overall model and 3 separate models for persons aged 15–30 (younger group), 30–54 (midlife group), and 55 and older (older group). The age cut-offs were chosen to allow comparability with other research, particularly on elderly persons. Bonferroni correction was used to account for multiple testing, i.e. empirical *p*-values were multiplied by 4.

To test for an overall social gradient in disability, we used the models described above to predict the average number of impairments, pain problems, and A&P limitations in the four groups of social class. To correct for multiple testing, 99% confidence intervals were used. Sampling weights provided by the Federal Statistics Office were applied to the data. The variables used for the construction of the social class index were treated as observed, and all other variables were set to the class mean. Average marginal counts were computed using Stata's post-estimation command *margins* [35].

Across all study variables, less than 1% of values were missing, corresponding to 2,687 cases with one or more missing values. Proportions of missing values ranged from zero (gender, age) to 6% (income). In order to avoid list-wise exclusion of cases with some missing values, all missing values were imputed using the multiple imputation procedure MissForest [36]. While we report results from the imputed data set, the un-imputed data set was used in a sensitivity analysis and the results compared; no relevant differences in parameters were found.

All analyses were controlled for age in years (to account for variation in age within the age groups) and health-related behaviours (alcohol consumption, smoking and low physical activity). We initially considered two economic macro-level variables, the Gini coefficient and gross domestic product per Canton. Since both variables failed to improve model fit, we decided to exclude them from the analyses.

## Results

The study population is described in Table 1. More than two thirds of the Swiss population experience at least one type of impairment or pain. Around 16% experience at least one A&P limitation, with growing proportions in older age groups (Pearson's *r* = 0.25; *p*<0.0001).

Results of the mixed effects regressions are presented in Tables 2 and 3. Based on Pseudo R^2^ values, the best overall model fit was obtained for the model explaining A&P limitation, followed by perceived health, impairment, and pain. The regression models fitted the data better in the midlife and older age group than in the younger group.

**Table 2:** Standardized parameters (betas) of mixed effects Poisson regressions of impairment and pain indices on selected independent variables.

Independent variables	Impairment				Pain			
	Total	≤30 years	31–54 years	≥55 years	Total	≤30 years	31–54 years	≥55 years
*Functioning*								
Pain	53.17***	14.58***	34.31***	37.37***	n.a.	n.a.	n.a.	n.a.
Impairment	n.a.	n.a.	n.a.	n.a.	54.01***	15.88***	35.62***	37.03***
*Socio-economic status*								
Male	−8.74***	−5.11***	−6.88***	−3.38***	−11.79***	−7.49***	−4.53***	−8.18***
Paid employment	−5.36***	**−0.45**	−3.77***	**−2.85** *	0.13	2.78*	**−0.20**	−3.38**
Years of formal education	−4.83***	**−0.55**	**−2.34**	−5.11***	**−1.75**	0.16	**−2.89** *	0.56
Income	**−1.86**	**−0.63**	0.47	**−2.21**	**−2.40**	**−1.06**	**−1.90**	**−1.51**
Migration background	**1.15**	**0.70**	**0.54**	**0.26**	**−**0.25	**−**0.58	**−**0.01	**−**0.45
*Social integration*								
Social support	−19.64***	**−1.32**	−13.59***	−11.57***	−12.52***	−5.56***	−7.74***	−7.36***
Social network utilization	−5.77***	−6.00***	**−2.34**	−4.65***	**−0.12**	**−0.19**	**−0.65**	0.24
Married	**−2.52** *	**−1.01**	**−2.74** *	**−0.84**	4.78***	**−0.25**	2.67*	1.76
Intercept	6.91***	*1.58*	4.72***	*0.95*	11.02***	5.19***	6.05***	9.81***
N	18,760	3,172	7,966	7,622	18,760	3,172	7,966	7,622
Pseudo R^2^	0.10	0.07	0.09	0.10	0.07	0.05	0.07	0.07

* Bonferroni corrected (p*4) probabilities *p<0.05, **p<0.01, ***p<0.001; parameters pointing in the direction of hypotheses (Figure 1) are in bold.

Note: all models have been adjusted for age and health-related behaviours (alcohol consumption, smoking, physical activity)

**Table 3:** Standardized parameters (betas) of mixed effects Poisson and Ordinal Probit regression models of activity and participation limitations and general health perception on selected independent variables.

Independent variables	Activity & participation limitation				Perceived health			
	Total	≤30 years	31–54 years	≥55 years	Total	≤30 years	31–54 years	≥55 years
*Functioning*								
Pain	16.53***	**3.01** *	12.00***	13.58***	−25.38***	−5.94***	−15.91***	−18.26***
Impairment	50.28***	14.00***	22.94***	41.01***	−22.47***	−7.02***	−12.51***	−16.58***
Activity & participation limitation	n.a.	n.a.	n.a.	n.a.	−26.56***	−4.70***	−13.81***	−22.36***
*Socio-economic status*								
Male	17.39***	5.98***	7.51***	12.04***	−7.82***	1.80	−4.15***	−9.82***
Paid employment	−33.74***	−8.48***	−24.26***	−14.00***	8.00***	**1.72**	6.00***	9.20***
Years of formal education	−9.79***	−9.72***	−5.46***	−4.14***	4.72***	**0.88**	3.92***	4.27***
Income	−4.15***	**−2.77** *	**−2.02**	**−2.32**	5.46***	3.15***	**2.99** *	4.96***
Migration background	**1.30**	−0.21	**1.21**	−0.72	**−0.96**	0.39	**−1.14**	**−1.35**
*Social integration*								
Social support	3.23*	**−1.32**	**−1.89**	4.04***	7.58***	**2.77** *	4.72***	4.77***
Social network utilization	−19.61***	−6.00***	−4.61***	−17.82***	**2.23**	**1.82**	**0.41**	**1.81**
Married	−8.45***	**−1.01**	−5.15***	**−1.99**	−2.93*	**1.72**	**1.74**	−1.86
Intercept	−16.22***	4.24***	−5.36***	−23.73***	‡	†	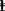	┐
N	18,760	3,172	7,966	7,622	18,760	3,172	7,966	7,622
Link	Poisson	Poisson	Poisson	Poisson	OProbit	OProbit	OProbit	Probit
Pseudo R^2^	0.34	0.22	0.33	0.30	0.15	0.06	0.12	0.16

* Bonferroni corrected (p*4) probabilities *p<0.05, **p<0.01, ***p<0.001; parameters pointing in the direction of hypotheses are printed in bold.

‡Intercepts (in standard units) were −43.48*** for the threshold between “very bad" and the rest; −35.18*** for “very bad" and “bad" vs. the rest; −21.60*** for “very bad", “bad", and “fair" vs. the rest; 10.51*** for all other categories vs. “very good".

†Intercepts (in standard units) were −12.09*** for the threshold between “very bad" and the rest; −12.98*** for “very bad" and “bad" vs. the rest; −8.86*** for “very bad", “bad", and “fair" vs. the rest; 3.99*** for all other categories vs. “very good".

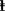
 Intercepts (in standard units) were −22.83*** for the threshold between “very bad" and the rest; −18.30*** for “very bad" and “bad" vs. the rest; −12.42*** for “very bad", “bad", and “fair" vs. the rest; 4.72*** for all other categories vs. “very good".

┐ Intercepts (in standard units) were −15.19*** for the threshold between “very bad" and the rest; −9.13*** for “very bad" and “bad" vs. the rest; −2.07 for “very bad", “bad", and “fair" vs. the rest; 10.61*** for all other categories vs. “very good". OProbit  =  Ordinal Probit.

Note: all models have been adjusted for age and and health behaviours (alcohol consumption, smoking, physical activity).

### Impairment

Male gender, paid employment, higher education, higher social support and social network utilization were all associated with a decreased number of reported impairments (Table 2). The number of pain problems was positively associated with the number of reported impairments. No significant effects of income or migration background on impairment were observed. Overall, results were consistent across age groups. Apart from social network utilization, all effects of these variables on impairment were more pronounced in the midlife and older groups (considering the betas and intercepts). The effect of ‘being married’ was confined to the midlife group.

### Pain

The number of pain problems decreased with perceived social support, but increased with the number of impairments (Table 2). Being married was associated with a higher number of pain problems. While being male reduced reported pain, education and paid employment did not strongly influence the number of reported pain problems in the overall model. Income and migration background again did not reach statistical significance. Higher education, however, reduced the number of reported pain problems in the midlife group. Being in paid employment increased the reported number of pain problems in the younger group, but decreased it in the older group. Perceived social support, in turn, exhibited a consistent, strong inverse relationship with number of pain problems across all age groups.

### A&P limitation

The number of A&P limitations increased among persons who reported more problems in pain and impairment (Table 3). Being in paid employment, having higher education or higher income was associated with lower levels of A&P limitation in the overall model. Migration background did not play a role. Stronger social network utilization was related to lower levels of A&P limitation, which was consistently observed across age groups. Stronger social support was associated with more A&P limitation in the overall model which seems to be due to the older group. Pain, impairment and SES variables (with the exception of income and migration background) displayed strong effects across all age groups. In contrast to impairment and pain, male gender was associated with less A&P limitation.

### Perceived health

A&P limitation, pain, and impairment were the strongest negative ‘predictors’ of perceived health, while being in paid employment and perceiving high levels of social support were the strongest positive ‘predictors’ in the overall model (Table 3). Higher education and income as well as social network utilization (Bonferroni corrected *p*-value  = 0.1) further contributed to better perceived health. Being married and being male were associated with worse perceived health in the overall model. These effects were particularly pronounced in the elder group. Migration background did not assert significant influence when it was adjusted for the other predictors.

### Overall social gradient of disability

**Figure 2:**
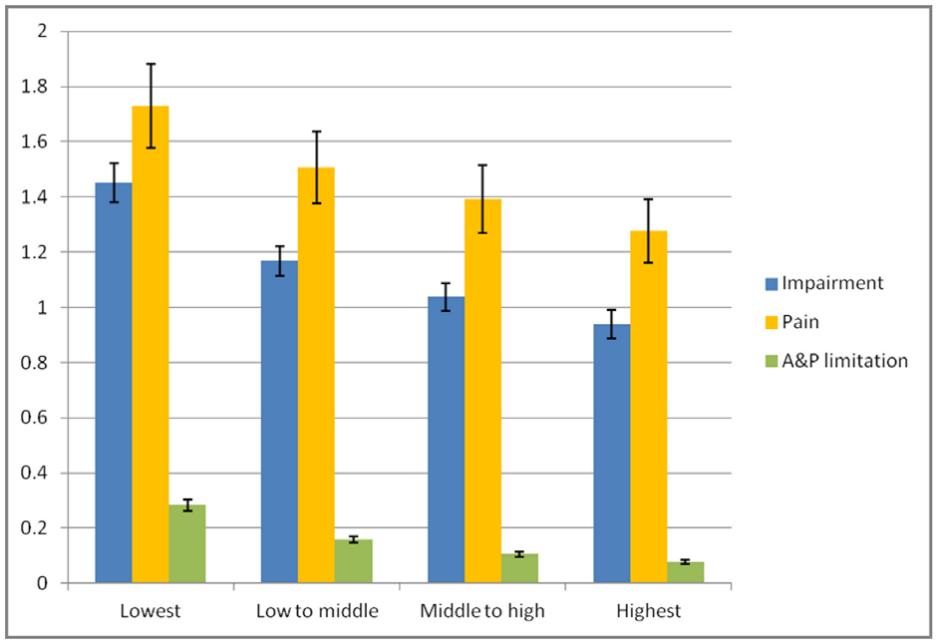
Predicted average number of problems in impairment, pain, and activity & participation (A&P) limitation by social class. Error bars represent 99% confidence intervals. Data are weighted with sampling weights and adjusted for age, gender, being married, employment, migration background, smoking, alcohol consumption and leisure physical activity. In addition, impairment is adjusted for pain, pain for impairment and A&P limitation for impairment and pain.

Figure 2 shows the numbers of problems in impairment, pain and A&P limitation across 4 social class groups based on the above models (marginal counts). The overall picture suggests a social gradient of disability in the Swiss general population with higher social class being associated with less disability.

## Discussion

In this cross-sectional study of a representative sample of the Swiss population, we found that measures of SES and social integration were strongly associated with several dimensions of disability and perceived health. Socially deprived and isolated people were at higher risk of disability. Conversely, the negative effects of impairments on pain, of pain on impairments, and of impairment and pain on activity and participation could be compensated by higher income, better education and social integration (Tables 2 and 3). To a large extent, this finding was consistent across all age groups. Migration background did not play a significant role when it was adjusted for other measures of SES and social integration. Gender, however, was among the strongest predictors of disability and perceived health, even when we adjusted for all other SES and social integration variables. Being male positively influenced impairment and pain but negatively affected A&P limitation and perceived health, when it was adjusted for the remaining disability components. This finding suggests differential response behaviours of men and women, potentially influenced by interactions of gender with other SES and social integration variables. To our knowledge, this is the first investigation that comprehensively explores potential social determinants of functioning and disability in a representative sample of young, middle-aged and older Swiss residents of both sexes. It also pioneers the application of the ICF framework for disability research using a large national database. Most of our findings were in agreement with our *a priori* hypotheses, thus supporting the ICF's multidimensional approach towards disability (Figure 1).

Our results are in line with studies from Greece [8] and Estonia [14] showing a social gradient of disability in the general population. They also support findings from previous studies reporting a social gradient of disability in the elderly [9], [10], [11], [12], [13], [18] but draw on more complex outcomes. In our study, most associations have the same direction but differ in strength across the age groups. These differences highlight the need for longitudinal studies in order to elucidate the evolution of disability patterns along the course of life. The hypotheses on SES and social integration were tested simultaneously, i.e. respective parameters were adjusted for each other. Consequently, our study confirms the findings from Nilsson [16] in middle-aged Danish men showing independent influence of financial assets and social support. Nonetheless, social support might compound the influences of SES on functioning and disability. We did, however, not examine respective interactions in our study.

Several limitations need to be taken into account. Firstly, our theoretical model assumes unidirectional effects. However, the cross-sectional design did not allow the direction of reported effects to be tested. Secondly, we did not elaborate on gender-specific associations. Nevertheless, we observed gender-specific effects and gender is known to interact with socioeconomic variables. A more detailed investigation would be desirable but was not the focus of this analysis. Thirdly, the variables relating functional status did not allow the severity of problems to be gauged. For instance, for pain, only the number of pain problems could be used without information on pain severity or duration. Fourthly, we used 4 separate multiple mixed effects regression models and did not perform a test of an overall model, e.g. based on structural equations [32] or conditional independence graphs [37], [38]. Further clarification of the structure of associations (Figure 1), including potential moderation and mediation [39], is required from future research. For example, it may be the case that the influence of impairment on A&P limitation is mediated or moderated by social position or gender. Finally, Switzerland is a high resource setting with high standards of health care and social welfare. This makes it difficult to generalize our findings to lower resourced settings where we would expect social inequalities to be even more pronounced, though typically data of good quality are not available from these settings.

These limitations are balanced by several strengths. Our results are derived from a representative sample of the Swiss population stratified into three age groups and the application of a comprehensive set of variables measuring functioning. Furthermore, we applied a theoretically-based approach to linking socioeconomic and sociological factors with newly constructed sum indices of functioning and with perceived health. Robust statistical methods included confirmatory factor analysis of the indices, mixed effects regression models to account for unobserved heterogeneity, Bonferroni correction to account for multiple testing, and tests of the models' fit to the data.

This study highlights consistent social inequalities in functioning and perceived health in a representative sample of the Swiss population aged 15 or above. Higher levels of social integration and of SES were associated with better functioning in terms of impairment, pain and limitations of A&P and better perceived health. Our findings are challenging in view of the high living standard in Switzerland. Indeed, given the social gradients in disability observed in this setting, they are likely to be even more pronounced elsewhere.
